# Supplementary material for: A Method for Quantifying Molecular Interactions Using Stochastic Modelling and Super-Resolution Microscopy
Source: Sci Rep. 2017 Nov 1;7:14882. doi: 10.1038/s41598-017-14922-8 (PMC5665986; doi:10.1038/s41598-017-14922-8)
Supplement: Supplementary file 1 — Supplementary Figures and Table [file 41598_2017_14922_MOESM1_ESM.pdf]

A Method for Quantifying Molecular Interactions Using Stochastic Modelling and Super-Resolution Microscopy

Supplementary Information

Keria Bermudez-Hernandez[1,2], Sarah Keegan[1,2], Donna R. Whelan[1], Dylan A. Reid[1], Jennifer Zagelbaum[1], Yandong Yin[1], Sisi Ma[2], Eli Rothenberg[1], David Fenyö[1,2]

[1]Dept. of Biochemistry and Molecular Pharmacology, New York University School of Medicine, New York, NY, USA

[2]Institute for Systems Genetics, New York University School of Medicine, New York, NY, USA

Keria Bermudez-Hernandez and Sarah Keegan contributed equally to this work.

Correspondence should be addressed to David Fenyö (david@fenyolab.org) or Eli Rothenberg (eli.rothenberg@nyumc.org).

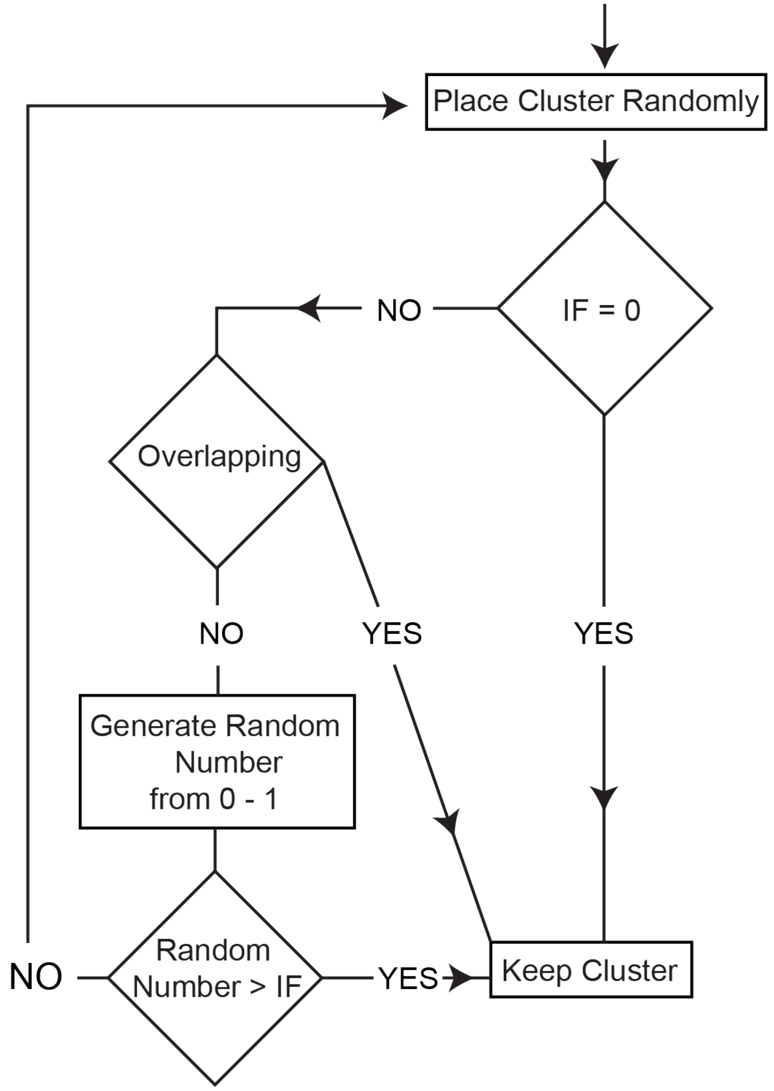

Supplementary Figure 1

Flowchart of algorithm to generate IF simulation. First, clusters of one color are placed randomly on the image. Then, clusters of the second color (reference color) are placed according to the flowchart. Workflow is repeated for each cluster in the experimental image.

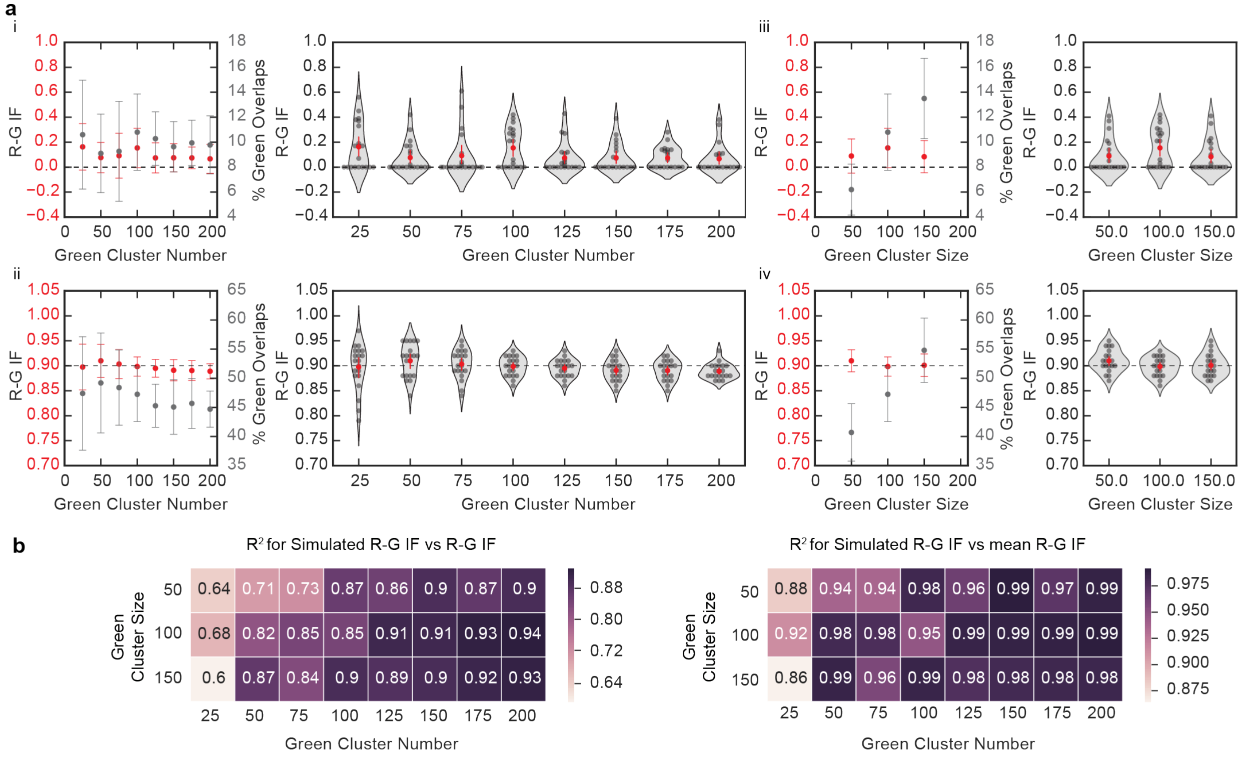

Supplementary Figure 2

Effects of increasing the size and number of green clusters in the calculation of R-G IF

(a) Plots of the percentage of green overlaps (gray) and calculated R-G IF (red) for simulations generated with increasing green cluster number for R-G IF = 0 (i) and IF = 0.90 (ii). (i) Simulations for R-G IF = 0 show that the percentage of green overlaps (Kruskal-Wallis H test: p = 0.60) and R-G IFs (Kruskal-Wallis H test: p = 0.35) remain constant with increasing green cluster number. (ii) Simulations for R-G IF = 0.9 show that the percentage of green overlaps (Kruskal-Wallis H test: p = 0.24) and R-G IFs (Kruskal-Wallis H test: p = 0.08) remain constant with increasing green cluster number (means and SD: n = 20 images per cluster number). (iii-iv) Plots of the percentage of green overlaps (gray) and calculated R-G IF (red) for simulations generated with increasing green cluster size for R-G IF = 0 (iii) and R-G IF = 0.90 (iv). (iii) Simulations for R-G IF = 0 show that the percentage of green overlaps increases with increasing green cluster size (One-way ANOVA: p < 0.0001) while R-G IFs remain constant (One-way ANOVA: p = 0.23). (iv) Simulations for R-G IF = 0.9 show that the percentage of green overlaps increases with increasing green cluster size (One-way ANOVA: p < 0.0001) while R-G IFs remain constant (One-way ANOVA: p = 0.21; means and SD: n = 20 images per cluster number). (a)(i)-(iv) show that increasing green cluster number/size doesn’t affect the calculated R-G IF.

(b) Heat maps of the R^2 calculated from plotting the simulated R-G IF vs calculated R-G IF for simulations with different green cluster number and sizes show that the error range is greater in images with low green cluster number and size (left; n = 20 images per IF). Heat maps of the R^2 calculated from plotting the mean simulated R-G IF vs mean calculated R-G IF for the same simulations as left (right; n = 20 images per IF).

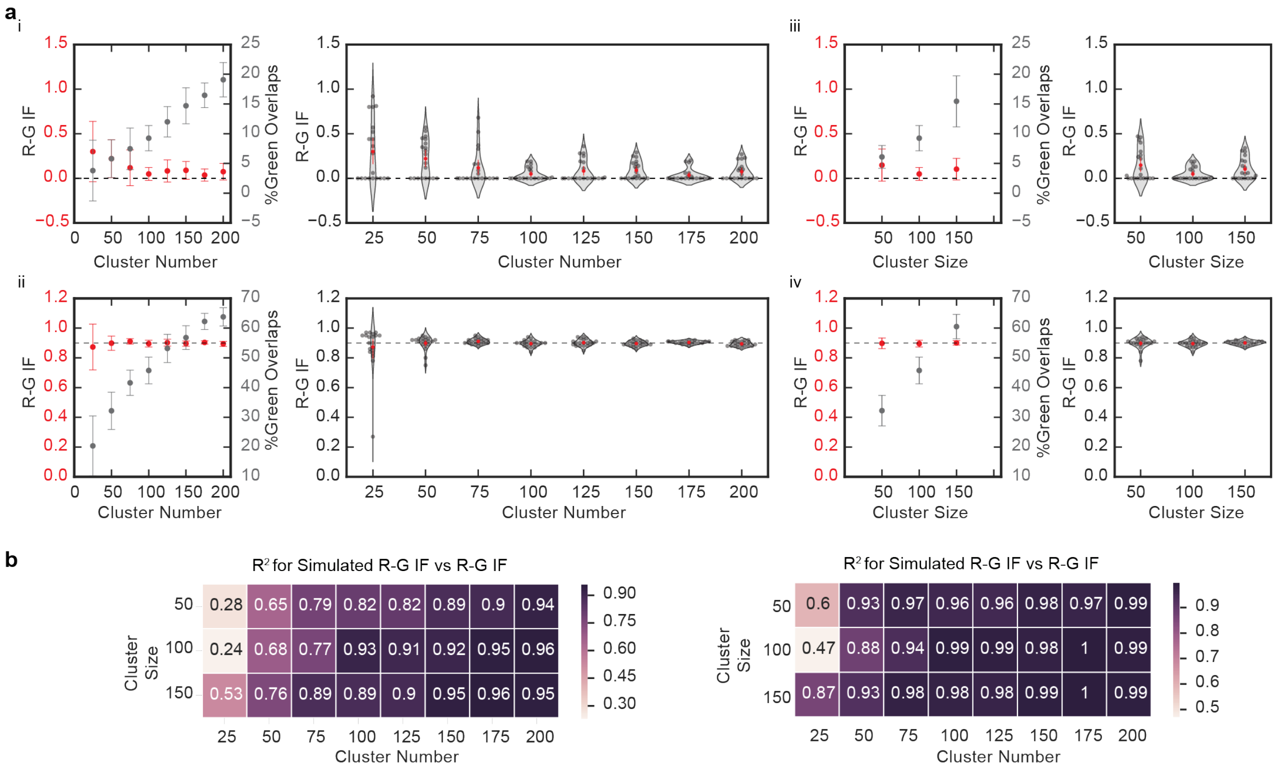

Supplementary Figure 3

Effects of increasing the size and number of red and green clusters in the calculation of R-G IF

(a) Plots of the percentage of green overlaps (gray) and calculated R-G IF (red) for simulations generated with increasing green and red cluster number for R-G IF = 0 (i) and R-G IF = 0.90 (ii). (i) Simulations for R-G IF = 0 show that the percentage of green overlaps increases with increasing cluster number (Kruskal-Wallis H test: $p < 0.0001$) while R-G IFs remain constant (Kruskal-Wallis H test: $p = 0.06$). (ii) Simulations for R-G IF = 0.90 show that the percentage of green overlaps increases with increasing cluster number (Kruskal-Wallis H test: $p < 0.0001$) while R-G IFs remain constant (Kruskal-Wallis H test: $p = 0.18$; means and SD: $n = 20$ images per cluster number). (iii-iv) Plots of the percentage of green overlaps (gray) and calculated R-G IF (red) for simulations generated with increasing green and red cluster size for R-G IF = 0 (iii) and R-G IF = 0.90 (iv). (iii) Simulations for R-G IF = 0 show that the percentage of green overlaps increases with increasing cluster size (Kruskal-Wallis H test: $p < 0.0001$) while R-G IFs remai-n constant (Kruskal-Wallis H test: $p = 0.20$). (iv) Simulations for R-G IF = 0.90 show that the percentage of green overlaps increases with increasing cluster size (One-way ANOVA: $p < 0.0001$) while R-G IFs remain constant (Kruskal-Wallis H test: $p = 0.54$; means and SD: $n = 20$

images per cluster number). (a)(i)-(iv) show that increasing both green and red cluster number/size doesn't affect the calculated R-G IF.

(b) Heat maps of the R^2 calculated from plotting the simulated R-G IF vs calculated R-G IF for simulations with different cluster number and sizes show that the error range is greater in images with low cluster number and size (left; n = 20 images per R-G IF). Heat maps of the R^2 calculated from plotting the mean simulated R-G IF vs mean calculated R-G IF for the same simulations as left (right; n = 20 images per R-G IF).

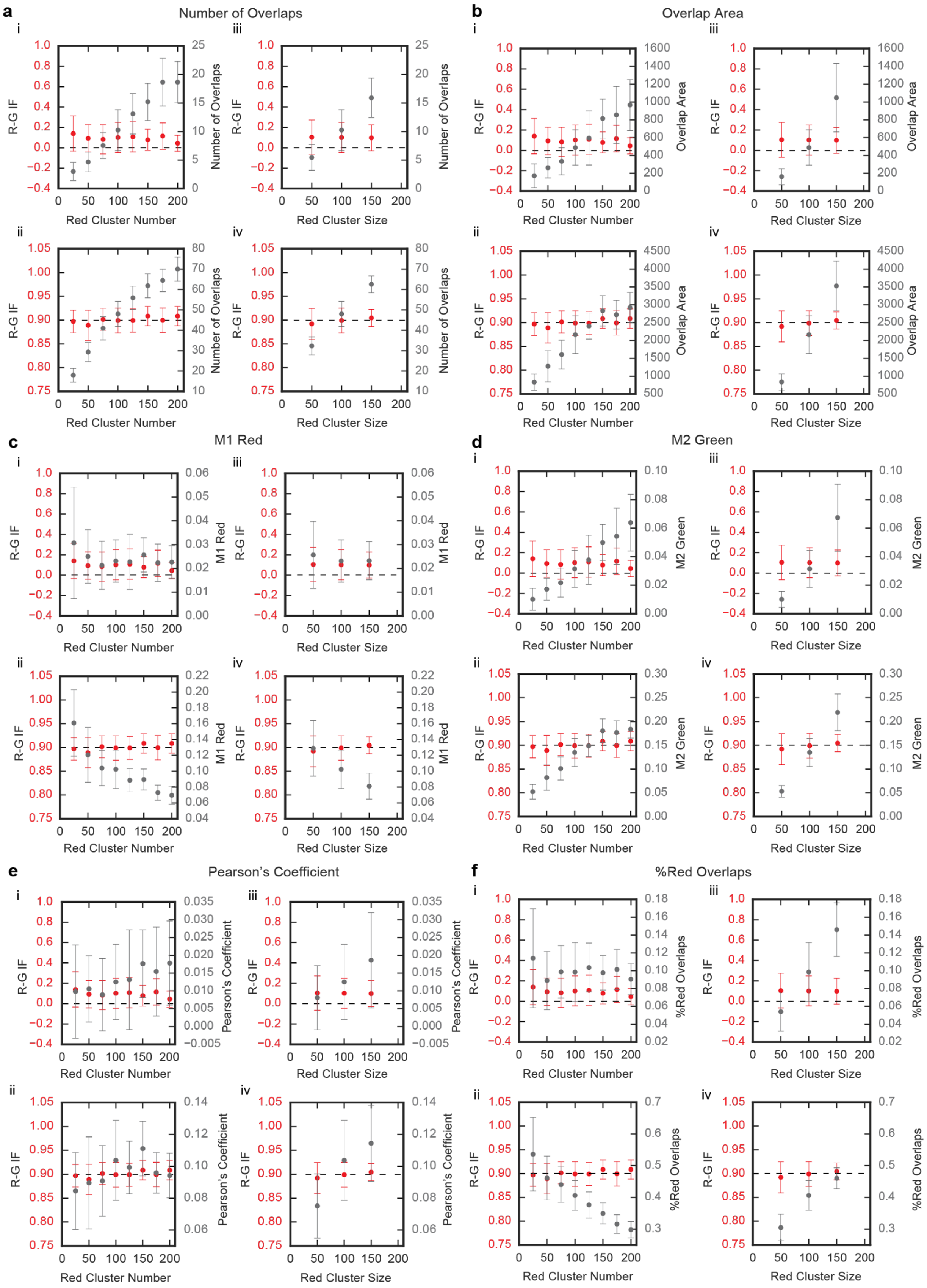

a
Number of Overlaps
i
ii
iii
iv
R-G IF
Number of Overlaps
Red Cluster Number
Red Cluster Size
b
Overlap Area
Overlap Area
c
M1 Red
M1 Red
d
M2 Green
M2 Green
e
Pearson's Coefficient
Pearson's Coefficient
f
%Red Overlaps
%Red Overlaps

Supplementary Figure 4

Comparison of the R-G IF with other Measures

(a) Plots of the number of overlaps (gray) and calculated R-G IF (red) for simulations generated with increasing red cluster number for R-G IF = 0 (i) and R-G IF = 0.90 (ii). (i) Simulations for R-G IF = 0 show that the number of overlaps increases with increasing red cluster number (Kruskal-Wallis H test: $p < 0.0001$) while R-G IFs remain constant. (ii) Simulations for R-G IF = 0.90 show that the number of overlaps increases with increasing red cluster number (One-way ANOVA: $p < 0.0001$) while R-G IFs remain constant (means and SD: n = 20 images per cluster number). (iii-iv) Plots of the number of overlaps (gray) and calculated R-G IF (red) for simulations generated with increasing red cluster size for R-G IF = 0 (iii) and R-G IF = 0.90 (iv). (iii) Simulations for R-G IF = 0 show that the number of overlaps increases with increasing red cluster size (One-way ANOVA: $p < 0.0001$) while R-G IFs remain constant. (iv) Simulations for R-G IF = 0.90 show that the number of overlaps increases with increasing red cluster size (One-way ANOVA: $p < 0.0001$) while R-G IFs remain constant (means and SD: n = 20 images per cluster size).

(b) Plots of the area of overlaps (gray) and calculated R-G IF (red) for simulations generated with increasing red cluster number for R-G IF = 0 (i) and R-G IF = 0.90 (ii). (i) Simulations for R-G IF = 0 show that the area of overlaps increases with increasing red cluster number (Kruskal-Wallis H test: $p < 0.0001$) while R-G IFs remain constant. (ii) Simulations for R-G IF = 0.90 show that the area of overlaps increases with increasing red cluster number (One-way ANOVA: $p < 0.0001$) while R-G IFs remain constant (means and SD: n = 20 images per cluster number). (iii-iv) Plots of the area of overlaps (gray) and calculated R-G IF (red) for simulations generated with increasing red cluster size for R-G IF = 0 (iii) and R-G IF = 0.90 (iv). (iii) Simulations for R-G IF = 0 show that the area of overlaps increases with increasing red cluster size (Kruskal-Wallis H test: $p < 0.0001$) while R-G IFs remain constant. (iv) Simulations for R-G IF = 0.90 show that the area of overlaps increases with increasing red cluster size (Kruskal-Wallis H test: $p < 0.0001$) while R-G IFs remain constant (means and SD: n = 20 images per cluster size).

(c) Plots of the Mander's coefficient 1 for the red overlaps (M1: gray) and calculated R-G IF (red) for simulations generated with increasing red cluster number for IF = 0 (i) and IF = 0.90 (ii). (i) Simulations for R-G IF = 0 show that M1 remains constant with increasing red cluster number (Kruskal-Wallis H test: $p = 0.79$). (ii) Simulations for R-G IF = 0.90 show that M1 decreases with increasing red cluster number (Kruskal-Wallis H test: $p < 0.0001$) while R-G IFs remain constant (means and SD: n = 20 images per cluster number). (iii-iv) Plots of M1 (gray) and predicted R-G IF (red) for simulations generated with increasing red cluster size for R-G IF = 0 (iii) and R-G IF = 0.90 (iv). (iii) Simulations for R-G IF = 0 show that M1 remains constant with increasing red cluster size (Kruskal-Wallis H test: $p = 0.75$). (iv) Simulations for R-G IF = 0.90 show that M1 decreases with increasing red cluster size (Kruskal-Wallis H test: $p < 0.0001$) while R-G IFs remain constant (means and SD: n = 20 images per cluster size).

(d) Plots of the Mander’s coefficient 2 for the green overlaps (M2: gray) and calculated R-G IF (red) for simulations generated with increasing red cluster number for R-G IF = 0 (i) and R-G IF = 0.90 (ii). (i) Simulations for R-G IF = 0 show that M2 increases with increasing red cluster number (Kruskal-Wallis H test: $p < 0.0001$) while R-G IFs remain constant. (ii) Simulations for IF = 0.90 show that M2 increases with increasing red cluster number (One-way ANOVA: $p < 0.0001$) while R-G IFs remain constant (means and SD: n = 20 images per cluster number). (iii-iv) Plots of M2 (gray) and calculated R-G IF (red) for simulations generated with increasing red cluster size for R-G IF = 0 (iii) and R-G IF = 0.90 (iv). (iii) Simulations for R-G IF = 0 show that M2 increases with increasing red cluster size (Kruskal-Wallis H test: $p < 0.0001$) while R-G IFs remain constant. (iv) Simulations for R-G IF = 0.90 show that M2 increases with increasing red cluster size (Kruskal-Wallis H test: $p < 0.0001$) while R-G IFs remain constant (means and SD: n = 20 images per cluster size).

(e) Plots of the Pearson’s coefficients (gray) and calculated R-G IF (red) for simulations generated with increasing red cluster number for R-G IF = 0 (i) and R-G IF = 0.90 (ii). (i) Simulations for R-G IF = 0 show that the Pearson’s coefficients remain constant with increasing red cluster number (One-way ANOVA: $p = 0.09$). (ii) Simulations for R-G IF = 0.90 show that the Pearson’s coefficients change with increasing red cluster number (Kruskal-Wallis H test: $p = 0.02$) while R-G IFs remain constant (means and SD: n = 20 images per cluster number). (iii-iv) Plots of the Pearson’s coefficients (gray) and calculated R-G IF (red) for simulations generated with increasing red cluster size for R-G IF = 0 (iii) and R-G IF = 0.90 (iv). (iii) Simulations for R-G IF = 0 show that the Pearson’s coefficients increase with increasing red cluster size (One-way ANOVA: $p = 0.02$) while R-G IFs remain constant. (iv) Simulations for R-G IF = 0.90 show that the Pearson’s coefficients increase with increasing red cluster size (One-way ANOVA: $p < 0.0001$) while R-G IFs remain constant (means and SD: n = 20 images per cluster size).

(f) Plots of the percentage of red overlaps (gray) and calculated R-G IF (red) for simulations generated with increasing red cluster number for R-G IF = 0 (i) and R-G IF = 0.90 (ii). (i) Simulations for R-G IF = 0 show that the percentage of red overlaps remains constant with increasing red cluster number (Kruskal-Wallis H test: $p = 0.58$). (ii) Simulations for R-G IF = 0.90 show that the percentage of red overlaps decreases with increasing red cluster number (Kruskal-Wallis H test: $p < 0.0001$) while R-G IFs remain constant (means and SD: n = 20 images per cluster number). (iii-iv) Plots of the percentage of red overlaps (gray) and calculated R-G IF (red) for simulations generated with increasing red cluster size for R-G IF = 0 (iii) and R-G IF = 0.90 (iv). (iii) Simulations for R-G IF = 0 show that the percentage of red overlaps increases with increasing red cluster size (One-way ANOVA: $p < 0.0001$) while R-G IFs remain constant. (iv) Simulations for R-G IF = 0.90 show that the percentage of red overlaps increases with increasing red cluster size (One-way ANOVA: $p < 0.0001$) while R-G IFs remain constant (means and SD: n = 20 images per cluster size).

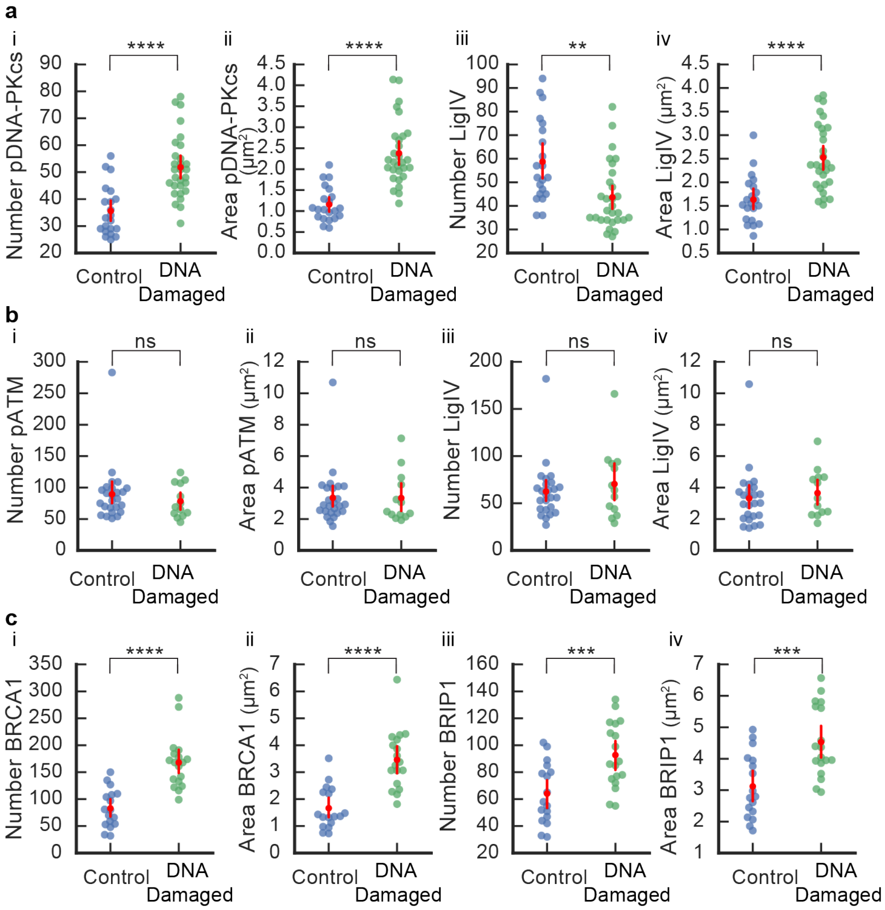

Supplementary Figure 5

Additional measurements for experimental datasets

(a) Number and total area covered by pDNA-PKcs and LigIV clusters. (i) The number of pDNA-PKcs clusters was greater in the DNA-damaged compared to control group (****$p<0.0001$; Student's t-test). (ii) The total area of pDNA-PKcs clusters was greater in the DNA-damaged compared to control group (****$p<0.0001$; Welch's t-test). (iii) The number of LigIV clusters was greater in the control compared to DNA-damaged group (**$p<0.01$; Student's t-test). (iv) The total area of LigIV clusters was greater in the DNA-damaged compared to control group (****$p<0.0001$; Student's t-test). Groups: control (n = 21) and DNA-damaged group (n = 29). Error bars represent 95 CI.

(b) Number and total area covered by pATM and LigIV clusters. (i) The number of pATM clusters in the control group was not significantly different from the DNA-damaged group (n.s. = not significant; $p>0.05$; Student's t-test) (ii) The total area of pATM clusters was not significantly different in the control compared to DNA-damaged group (n.s. = not significant; $p>0.05$;

Student's t-test). (iii) The number of LigIV clusters was not significantly different in the control compared to DNA-damaged group (n.s. = not significant; p>0.05; Student's t-test). (iv) The total area of LigIV clusters was not significantly different in the control compared to DNA-damaged group (n.s. = not significant; p>0.05; Student's t-test). Groups: control (n = 25) and DNA-damaged group (n = 13). Error bars represent 95 CI.

(c) Number and total area covered by BRCA1 and BRIP1 clusters. (i) The number of BRCA1 clusters was greater in the DNA-damaged compared to control group (****p<0.0001; Student's t-test). The total area covered by BRCA1 clusters was greater in the DNA-damaged compared to control group (****p<0.0001; Student's t-test). The number of BRIP1 clusters was greater in the DNA-damaged compared to control group (***p<0.001; Student's t-test). The total area covered by BRIP1 clusters was greater in the DNA-damaged compared to control group (***p<0.001; Student's t-test). Groups: control (n = 17) and DNA-damaged group (n = 18). Error bars represent 95 CI.

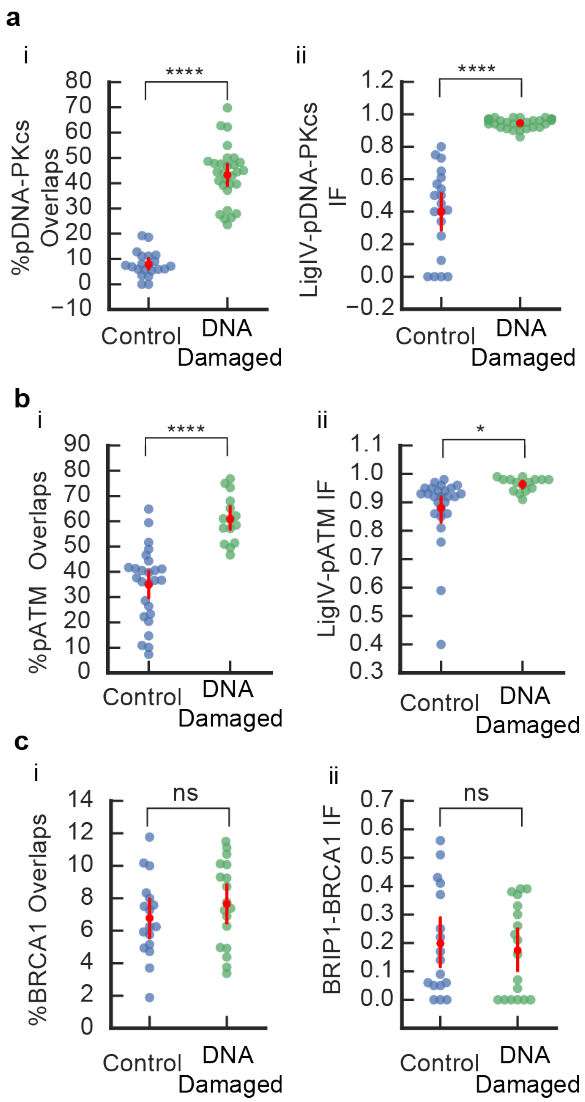

Supplementary Figure 6

Percentage of Overlaps and IF measurements when reference color is changed in experimental datasets

(a) LigIV and pDNA-PKcs measurements. (i) Percentage of pDNA-PKcs clusters overlapping with LigIV was greater in the DNA-damaged compared to control group (****p<0.0001; Welch's t-test). (ii) Interaction Factor (IF) between LigIV/pDNA-PKcs in control and DNA-damaged group. The IF between LigIV/pDNA-PKCcs was greater in the DNA-damaged compared to control group (****p<0.0001; Welch's t-test). Groups: control (n = 21) and DNA-damaged group (n = 29). Error bars represent 95 CI.

(b) LigIV and pATM measurements. (i) Percentage of pATM clusters overlapping with LigIV was greater in the DNA-damaged compared to control group (****$p<0.0001$; Student's t-test). (ii) Interaction Factor (IF) between LigIV/pATM in control and DNA-damaged group. The IF between LigIV/pATM was greater in the DNA-damaged compared to control group (*$p<0.05$; Welch's t-test). Groups: control (n = 25) and DNA-damaged group (n = 13). Error bars represent 95 CI.

(c) BRIP1 and BRCA1. (i) Percentage of BRCA1 clusters overlapping with BRIP1 was not significantly different in the DNA-damaged compared to control group (n.s = not significant; $p>0.05$; Student's t-test). (ii) Interaction Factor (IF) between BRIP1/BRCA1 in control and DNA-damaged group. The IF between BRIP1/BRCA1 was not significantly different in the DNA-damaged compared to control group (n.s. = not significant; $p>0.05$; Student's t-test). Groups: control (n = 17) and DNA-damaged group (n = 18). Error bars represent 95 CI.

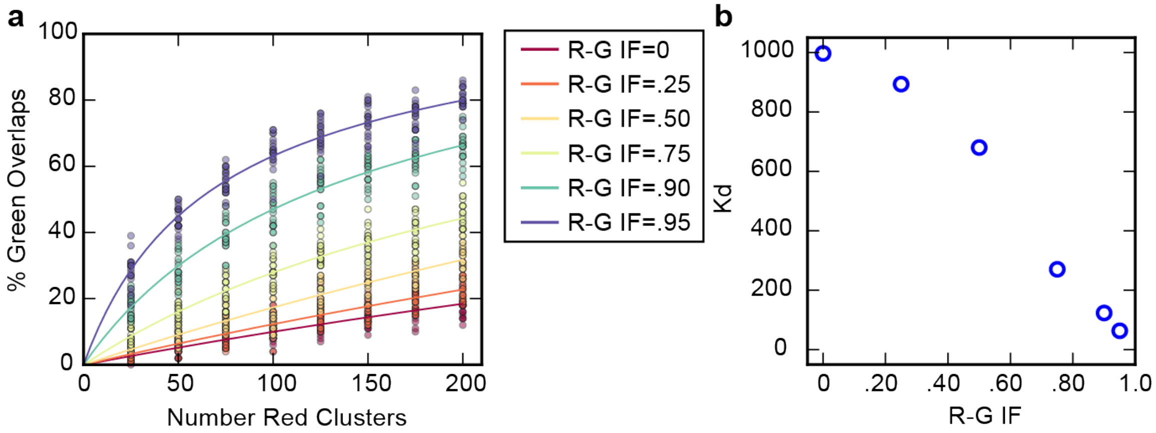

Supplementary Figure 7

Comparison of R-G IF with Kd

(a) Plot showing percentage of green cluster overlaps for simulations generated with different R-G IFs and red cluster number. Curves represent the function *y =100*x/(Kd+X) + NS*x* fitted to the points; points indicate values for individual simulations (n = 20 simulations per R-G IF).

(b) Plot of R-G IF vs Kd; Kd values were calculated from (a). A linear regression was fitted and resulted in R^2 = 0.97.

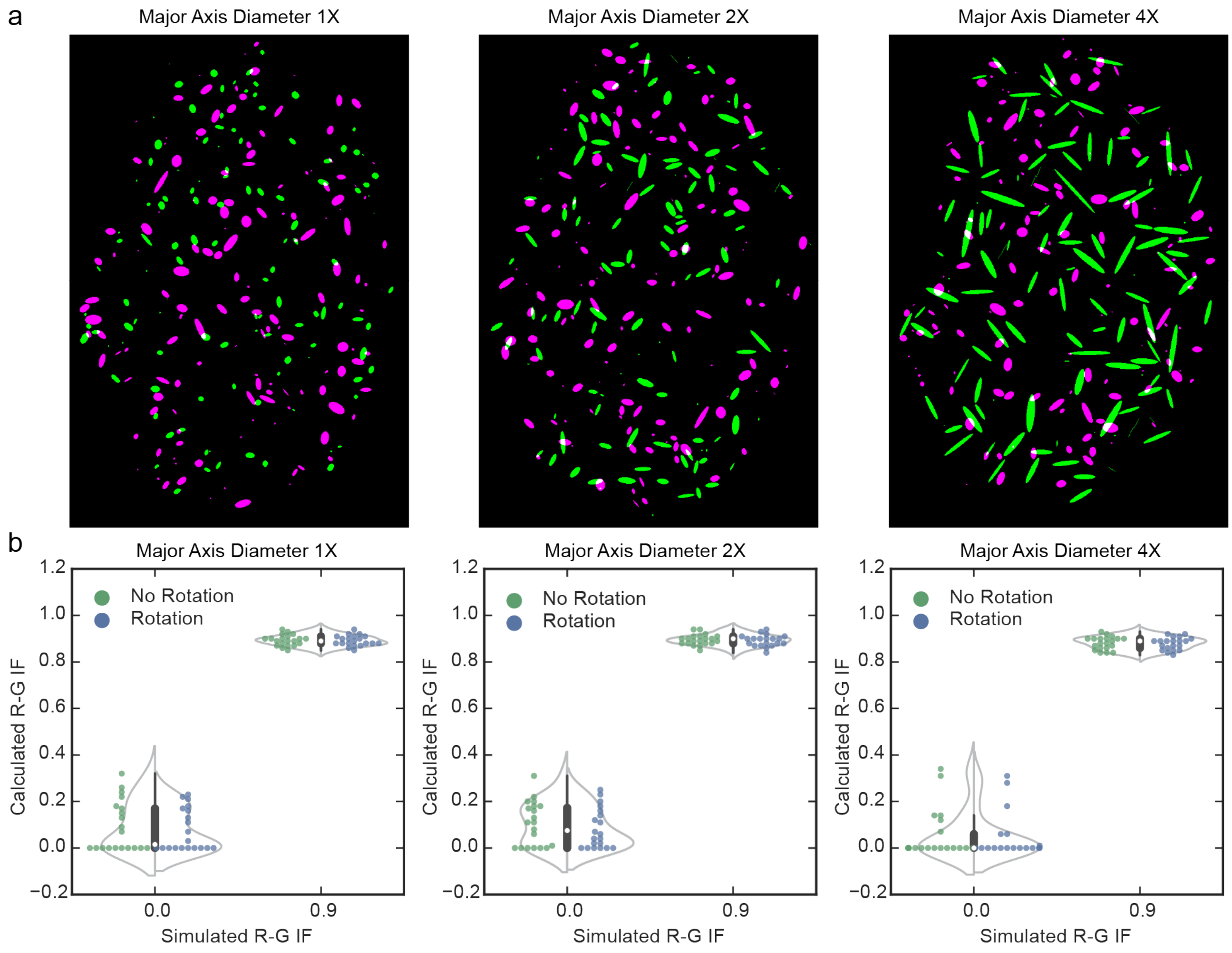

Supplementary Figure 8

Comparison of calculation of R-G IF with and without rotation for clusters with increasing major axis diameter

(a) Examples of simulations generated with 100 red clusters and100 green clusters with different major axis diameter. For illustration purposes, the color red is represented as magenta. In generating the simulated images, cluster numbers and size distributions were sampled from experimental image and the major axis diameter was multiplied by 1 (1x), 2 (2X), or 4 (4X).

(b) Plot of calculated R-G IF with and without rotation of clusters with increasing major axis diameter for simulations generated with R-G IF 0.0 and 0.9 (n = 20 simulations per R-G IF).

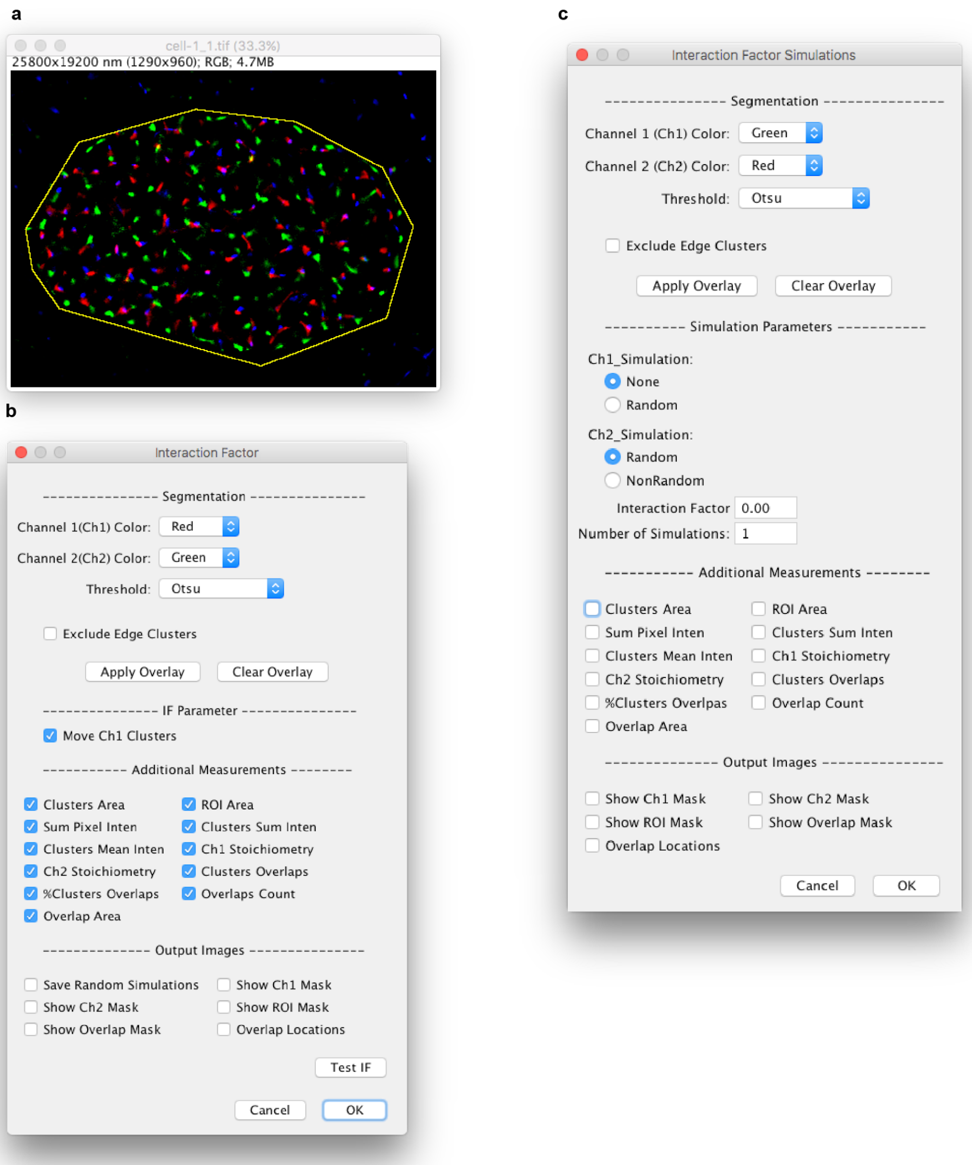

Supplementary Figure 9

Screenshots of Interaction Factor Package plugins

(a) Screenshot of super resolution image (dSTORM) of nucleus opened in ImageJ. ROI is outlined by the user in yellow.
(b) Screenshot of Interaction Factor ImageJ plugin.
(c) Screenshot of Interaction Factor Simulations ImageJ plugin.

Supplementary Table 1

IF	Range of Calculated IF
0	0.10
0.10	0.06
0.14	0.14
0.18	0.12
0.22	0.09
0.26	0.13
0.30	0.07
0.34	0.11
0.38	0.12
0.42	0.07
0.46	0.09
0.50	0.05
0.54	0.10
0.58	0.08
0.62	0.06
0.66	0.04
0.70	0.03
0.74	0.06
0.78	0.05
0.82	0.03
0.86	0.03
0.90	0.03
0.94	0.01
0.98	0.00

Table of range of variation in the calculated IF for images generated for IF= 0 to IF = 0.98.

Interaction Factor Package ImageJ Plugin Documentation

Keria Bermudez-Hernandez and Sarah Keegan

April 10, 2017

Table of Contents

Introduction ... **1**

Installation ... **1**

How To Use the Interaction Factor Plugins ... **2**

Interaction Factor Plugin ... **2**

Segmentation ... *3*

IF Parameter ... *5*

Additional Measurements ... *5*

Output Options ... *5*

Running the Calculation and Interpreting the Results ... *7*

Running the First Calculation ... 7

Output Images and Locations Table ... 10

Running Multiple Calculations ... 12

The Final Run ... 12

Cancelling the Analysis ... 13

Tips for Using and Interpreting the IF ... *13*

Interaction Factor Simulations Plugin ... **14**

Running the Simulation or Simulations ... *15*

Output ... *16*

Introduction

This plugin provides a method for quantifying protein-protein interactions by using stochastic modeling of super-resolution fluorescence microscopy data (RGB images). The result is an unbiased measure of co-localization of protein clusters, independent of cluster density and comparable across images. Please refer to manuscript (REF) for a detailed description of the Interaction Factor.

Installation

Copy the .jar file into the plugins folder of ImageJ or Fiji. Close and open ImageJ/Fiji. The plugins will be found under **Analyze**.

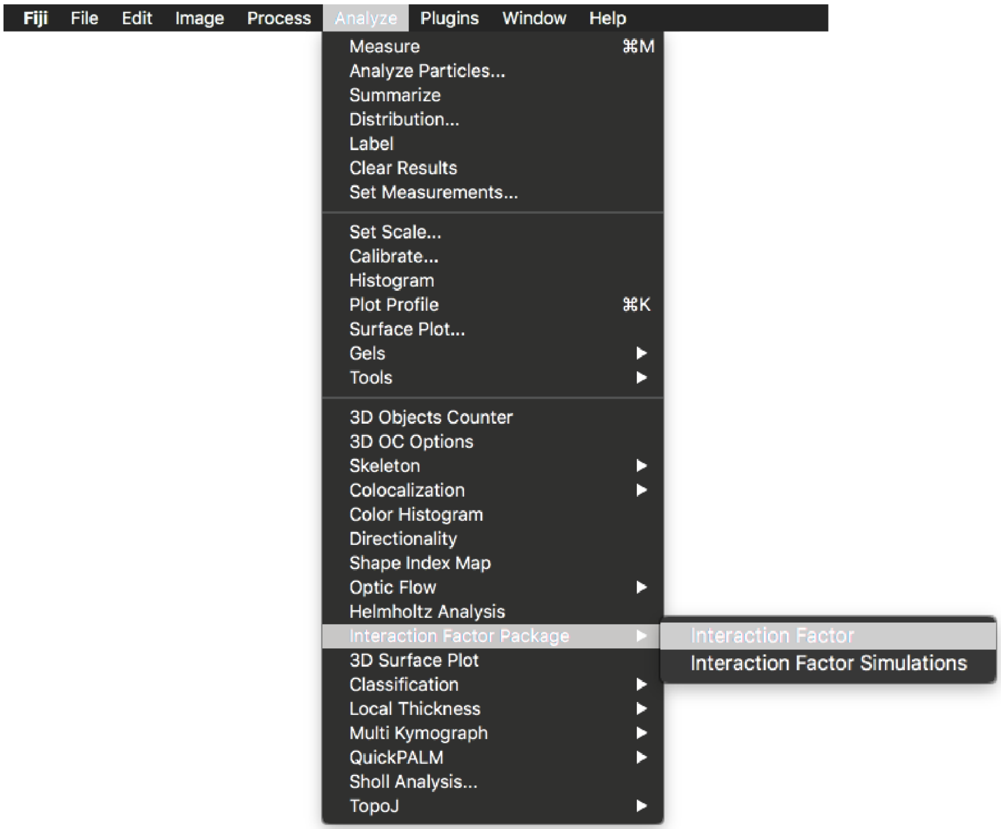

Figure 1. Interaction Factor Package

Important Installation Note: the plugin only works with Java8 and ImageJ v1.48 or newer. Download ImageJ bundled with Java8 here

How To Use the Interaction Factor Plugins

For both versions of the plugin, the first step is to start the Fiji or ImageJ application. Next, choose an image you wish to analyze. This is accomplished by using File/Open menu option or by dragging and dropping a file to the area under the Fiji toolbar. The next step is to choose the plugin which is found under the **Analyze** pull down menu. There are two versions of the plugin that may be used to analyze an image. The first version, called **Interaction Factor**, allows you to calculate the Interaction Factor for the image. The second version, called **Interaction Factor Simulations**, allows you to produce any number of simulations for the image at a user-defined IF for use in further analysis.

Interaction Factor Plugin

From the Interaction Factor menu selection, choose the first option – Interaction Factor. A pop up screen will appear (Figure 2).

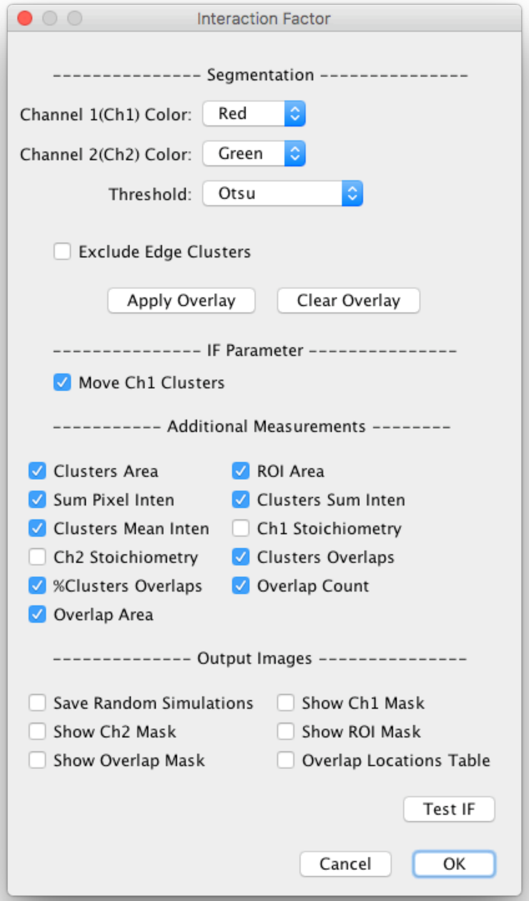

Figure 2. Interaction Factor Plugin

The first step is to select the Region of Interest (ROI) in the image by using one the four selection tools in the Fiji toolbar (Figure 3). Refer to this link for a tutorial on how to draw an ROI. If your region covers the field of view there is no need to draw an ROI. Next, there are a number of parameters that may be used as input to the analysis. They are found in the ***Segmentation*** and ***IF Parameter*** sections of the pop up window. In addition, there are a number of outputs that may be chosen for a given run. These are found in the ***Additional Measurements*** and ***Output Images*** sections of the pop up window.

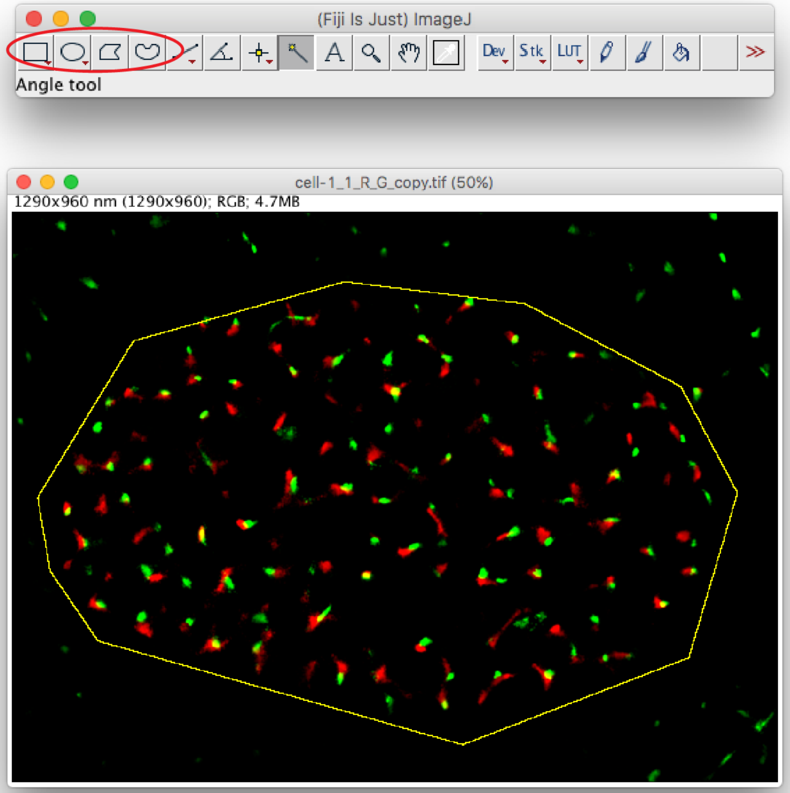

Figure 3. Example of ROI and selection tools. In red are the selection tools.

Segmentation

Here, you select the options for the chosen ROI you want to see in the analysis. These include the following:

1. **Channel 1(Ch1) Color**- from a pull down menu one of three colors may be chosen (Red, Blue,

Green) for the first channel

2. **Channel 2(Ch2) Color**- from a pull down menu one of three colors may be chosen (Red, Blue, Green) for the second channel
3. **Threshold**- from a pull down menu a threshold may be chosen. These are the standard ImageJ thresholds and additional information may be found at the ImageJ website.
4. **Exclude Edge Clusters**- the final options in this section is related to what information is included in the ROI. When selecting the ROI, the line may cut through a cluster. You may choose to include or not include those clusters that are not completely within the ROI.

Once all the options have been chosen, click the button *"**Apply Overlay**"*. All the clusters used in the analysis will be highlighted (in white) (Figure 4). If you wish to change any of the options then click the button *"**Clear Overlay**"*. The selected clusters will be deselected but the ROI will remain unchanged. You may then select new parameters in the ***Segmentation*** section.

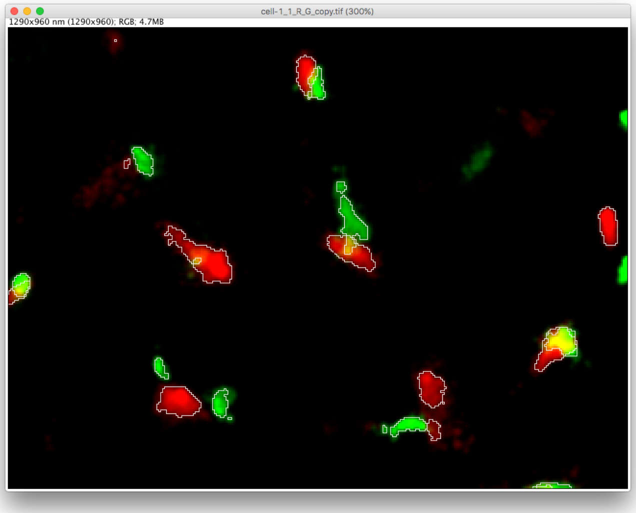

Figure 4. Example of zoomed-in image where clusters are outlined using the "**Apply Overlay**" button.

Note:

If the user has his or her own masks of the clusters created by another method, the user can still use the IF plugin by first combining the masks to an RGB image by going to **Image -> Color-> Merge Channels** and selecting channel 1 and channel 2 masks (make sure to uncheck the "**Make composite**" option). In the IF plugin select the Otsu threshold and continue to use the plugin normally.

IF Parameter

In this section, there is one parameter – **'Move Ch1 Clusters**' which is chosen by clicking the check the box (default). If this option is checked then, when calculating the IF, the simulations are going to be made such that Ch1 clusters will be placed randomly within the ROI. If the box is left unchecked, then Ch1 clusters will not be moved but stay in their current positions.

Additional Measurements

There are standard measurements displayed in the results table - the output from running an IF calculation. In addition to these, you may choose some other measurements you need to properly analyze your image.

1. **Clusters Area** - sum of the area of all the clusters multiplied by the square of the scale
2. **ROI Area**- is the sum of the area inside the ROI multiplied by the square of the scale
3. **Sum Pixel Inten**- is the sum of all the pixel intensities inside the ROI
4. **Clusters Sum Inten**- is the sum of all pixel intensities in the clusters
5. **Clusters Mean Inten** - is the mean intensities of all the clusters
6. **Ch 1 Stoichiometry**- is the percentage of Ch1 clusters that are overlapping with 1 (1:1), 2 (1:2), 3 (1:3), or > 3 Ch2 clusters (Stoichiometry)
7. **Ch 2 Stoichiometry**- is the percentage of Ch2 clusters that are overlapping with 1 (1:1), 2 (1:2), 3 (1:3), or > 3 Ch1 clusters (Stoichiometry)
8. **Clusters Overlaps**- is the number of clusters of one color overlapping with clusters with the opposite color
9. **%Clusters Overlaps** -is the percentage of clusters of one color overlapping with clusters of the opposite color
10. **Overlaps Count**- is the number of overlaps counted by first generating an overlap mask and counting the number of overlapping regions
11. **Overlap Area**- is calculated by adding the area of all the overlapping regions multiplied by the square of the scale

Note:
The scale is dependent on the resolution. For example, the scale for a super resolution image can represent 20nm. This number may be part of the metadata of the input image and is read by ImageJ when opening the image.

Output Options

There are several output options that may be selected as part of a run for further analysis of the results. Each option chosen will be displayed in a separate window. The possible selections include

the following:

1. **Save Random Simulations**- the 50 random images used in the simulations for calculating the IF.
2. **Show Ch1 Mask-** mask of Ch1 clusters within the ROI
3. **Show Ch2 Mask**- mask of the Ch2 clusters within the ROI
4. **Show ROI Mask**- mask of the entire ROI chosen by the selection tool
5. **Show Overlap Mask-** mask of overlap regions between Ch1 and Ch2
6. **Overlap Locations Table-** a table pertaining to the Overlap Mask. It shows the location, area, and number of pixels for each overlap in the mask.

Running the Calculation and Interpreting the Results

Running the First Calculation

Once all the options are selected, you can execute the calculation by clicking the "***Test IF***" button in the lower right. A results window will be displayed.

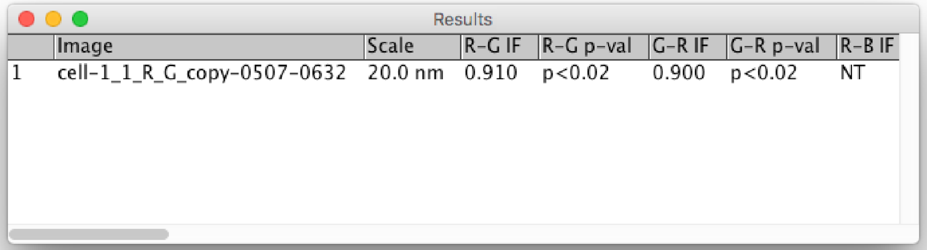

Results

	Image	Scale	R-G IF	R-G p-val	G-R IF	G-R p-val	R-B IF
1	cell-1_1_R_G_copy-0507-0632	20.0 nm	0.910	p<0.02	0.900	p<0.02	NT

Figure 5. Example of Results Table

The following is the data reported by the plugin:

1. **Image-** the number of the image used in the IF prediction. If there is an ROI ID, it will be added to the image name.
2. **Scale-** This is retrieved from the image meta data

For the next several columns, the IF and p-val are calculated with both Ch1 (Ch2-Ch1 IF) and Ch2 (Ch1-Ch2 IF) as the reference color. The reference color is the color for which the percentage of overlapping clusters is measured when calculating the IF (see manuscript for further explanation). A value of *"NT"* in the column value means *"Not Tested" (*these columns are included in case they are needed for future calculation).

3. **IF-** Interaction Factor between two color clusters is a number between 0 and 1 where 0 indicates no interaction and 1 indicates complete interaction. Below is a guide for understanding the IF. If the number is in the "no or low interaction level" refer to the p-value. If the p-value is <0.02 this means that its likely there is an interaction between the clusters. In other words, if the p-value is <0.02 there is less than 0.02 probability that the percentage of overlaps observed are due to random occurrence.

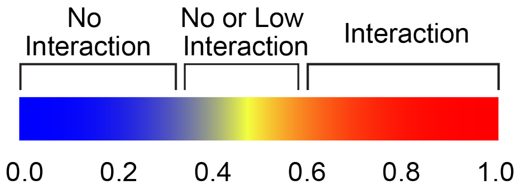

Figure 6. Guide for understanding the IF

4. **p-val-** the number of random simulations that had equal or greater percentage of overlapping clusters compared to the experimental divided by the total number of random simulations. In other words, if there were two random simulations that had equal or greater percentage of overlapping clusters compared to the experimental, then p-val = 2/50, = 0.04. If the p-value is <0.02 this means that none of the random simulations produced equal or greater percentage of overlapping clusters compared to the experimental image, and therefore it's likely there is an interaction between the clusters. In other words, if the p-value is <0.02 there is less than 0.02 probability that the percentage of overlaps observed are due to random occurrence.

For an RGB image where Ch1 and Ch2 are chosen by the user as red (R), green (G), or blue (B).

$$Ch1_{mask} = ROI_{mask} \cap (Ch1 > th1)$$

$$Ch2_{mask} = ROI_{mask} \cap (Ch2 > th2)$$

Where *th* is the threshold determined by the thresholding method selected from the dropdown menu and ROI_{mask} are the pixels inside the region of interest (ROI).

5. **Th Algorithm-** this is the algorithm chosen by the pull down menu in the ***Segmentation*** section
6. **R/G/B Th-** is the threshold determined by the thresholding method selected from the dropdown menu applied to the pixels inside the region of interest (ROI) for that channel
7. **R/G/B Clus Count-** count of all clusters in the corresponding Chi_{mask} (i=1,2)
8. **R/G/B Clus Area-** sum of the area of all the clusters multiplied by the square of the scale

$$Chi\ Clus\ Area = \sum Chi_{mask} \times scale^2$$

9. **ROI Area-** sum of the area inside the ROI multiplied by the square of the scale

$$ROI\ Area = \sum ROI_{mask} \times scale^2$$

10. R/G/B Sum Inten- sum of all the pixel intensities inside the ROI

$$Chi\ Sum\ Inten = \sum Chi \cap ROI_{mask}$$

11. R/G/B Clus Sum Inten- sum of all pixel intensities in the clusters

$$Chi\ Clus\ Sum\ Inten = \sum Chi_{mask} \cap Chi$$

12. R/G/B Clus Mean Inten- mean intensities of all the clusters

$$Chi\ Clus\ Mean\ Inten = \frac{\sum Chi_{mask} \cap Chi}{n}$$

where *n* is the number of pixels in *Chi* mask (clusters)

13. R/G/B Clus Overlaps- number of Ch2 clusters overlapping with Ch1 clusters and vice versa

14. R/G/B1:1, 1:2, 1:3, 1:>3- percentage of Ch2 clusters that are overlapping with 1 (1:1), 2 (1:2), 3 (1:3), or > 3 Ch1 clusters and vice versa (Stoichiometry)

15. R/G/B %Clus Overlaps- percentage of Ch2 clusters overlapping with Ch1 clusters and vice versa

16. Overlap Count- number of overlaps counted by first generating an overlap mask

$$Overlap_{mask} = Ch1_{mask} \cap Ch2_{mask}$$

and counting the number of overlapping regions.

17. Overlap Area- the area of all the overlapping regions multiplied by the square of the scale

$$Overlap\ Area = \sum Overlap_{mask} \times scale^2$$

Output Images and Locations Table

These are the examples of the optional output images

1. **Ch1 Mask**- mask of Ch1 clusters within the ROI

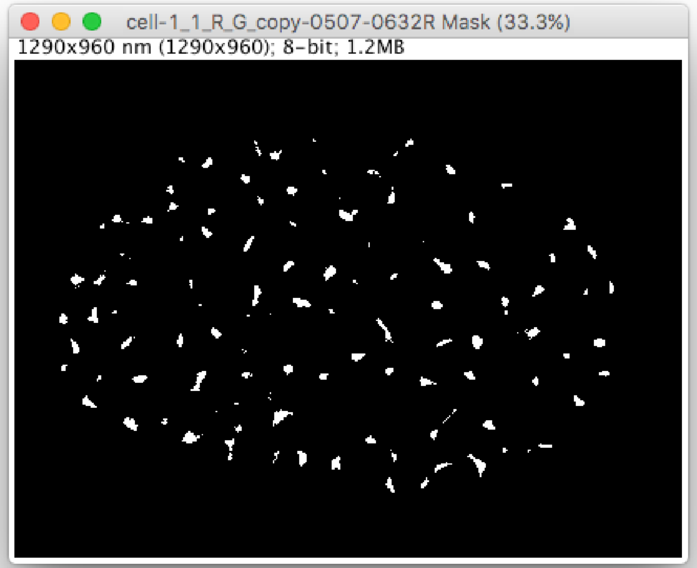

Figure 7. Example of Red Mask

2. **Ch2 Mask**- mask of the Ch2 clusters within the ROI

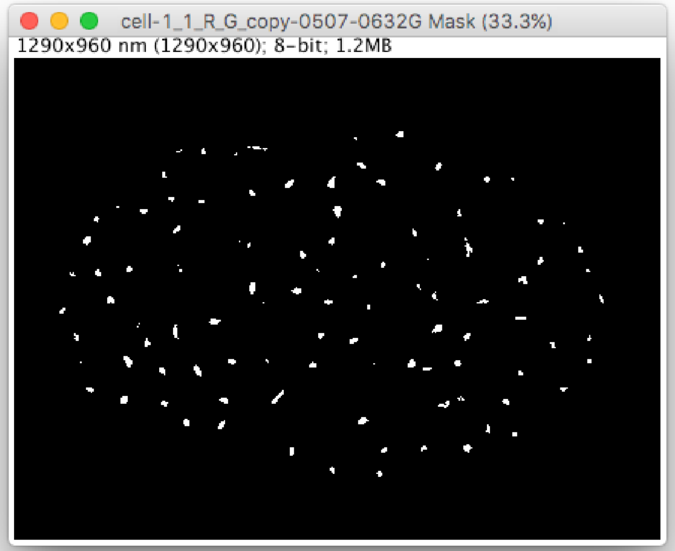

Figure 8. Example of Green Mask

3. **ROI Mask**- mask of the entire ROI chosen by the selection tool

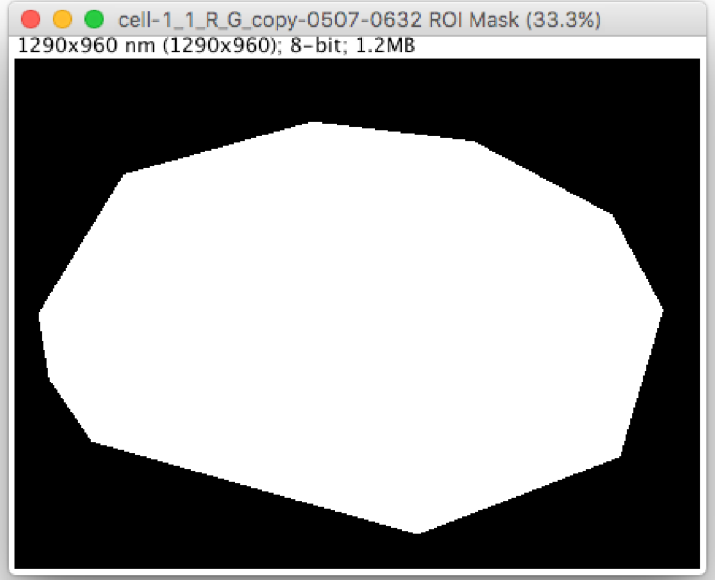

Figure 9. Example of ROI Mask

4. **Overlap Mask**- mask of overlap regions between Ch1 and Ch2

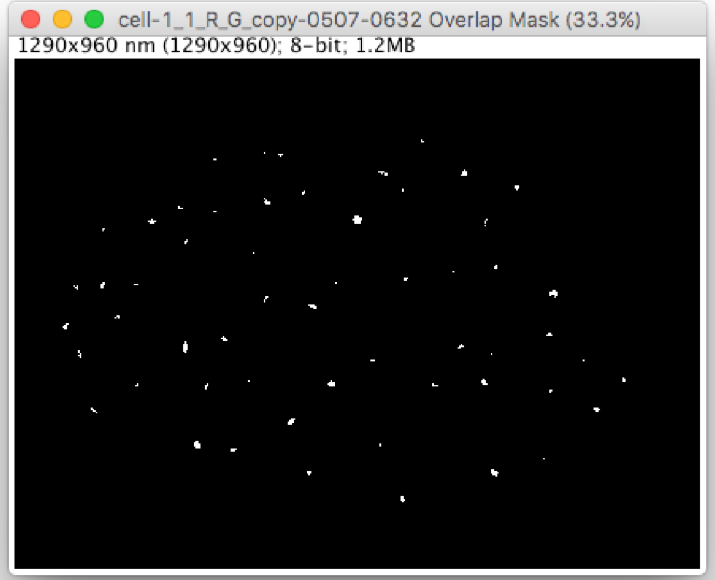

Figure 10. Example of Overlap Mask

5. **Overlap Locations Table-** a table pertaining to the Overlap Mask. It shows the location, area, and number of pixels for each overlap in the mask.
    a. **Image**-name of image
    b. **Number Pixels-** the number of pixels in the overlap
    c. **Area-** the number of pixels multiplied by the square of the scale
    d. **CentroidX-** x coordinate of the center of the overlap
    e. **CentroidY-** y coordinate of the center of the overlap

Overlap Locations

	Image	Number Pixels	Area	Centroid
1	cell-1_1_R_G_copy-0507-0632	88	35200	1855.00
2	cell-1_1_R_G_copy-0507-0632	45	18000	2274.88
3	cell-1_1_R_G_copy-0507-0632	44	17600	2405.00
4	cell-1_1_R_G_copy-0507-0632	57	22800	2905.08
5	cell-1_1_R_G_copy-0507-0632	84	33600	3252.61
6	cell-1_1_R_G_copy-0507-0632	27	10800	3282.59
7	cell-1_1_R_G_copy-0507-0632	38	15200	3826.31
8	cell-1_1_R_G_copy-0507-0632	21	8400	4507.14
9	cell-1_1_R_G_copy-0507-0632	19	7600	4570.00
10	cell-1_1_R_G_copy-0507-0632	74	29600	5135.67
11	cell-1_1_R_G_copy-0507-0632	30	12000	6197.33
12	cell-1_1_R_G_copy-0507-0632	151	60400	6387.48
13	cell-1_1_R_G_copy-0507-0632	42	16800	6427.14
14	cell-1_1_R_G_copy-0507-0632	145	58000	6843.51
15	cell-1_1_R_G_copy-0507-0632	44	17600	7193.18
16	cell-1_1_R_G_copy-0507-0632	28	11200	7494.28

Figure 11. Example of Overlap Locations Table

Running Multiple Calculations

You may execute multiple runs of the IF calculation with different input parameters and output images. Each run is stored in the results table as a separate row. None of the outputs are destroyed between runs but are accumulated. If any options are changed in the Segmentation and IF Parameter sections, it may be advisable to save one “type” of run in a separate folder for comparison with subsequent runs.

The Final Run

Up until now each calculation was executed by clicking the *“**Test IF**”* button which kept accumulating

all the output from the runs. Once all the analysis is completed a final calculation may be executed by clicking the "**OK**" button rather than the "**Test IF**" button. This closes the plugin pop-up screen. The original image and any output chosen remains displayed on the screen.

Cancelling the Analysis

You may end all of the analysis by clicking the "***Cancel***" button.

Tips for Using and Interpreting the IF

- It is possible that the value of the IF calculation will have a small variation for multiple runs of the same image since the calculation is based on the percentage of overlap from random images, which are created dynamically each time the IF is calculated. Please refer to the manuscript for further details (REF).
- The user should avoid the IF for images with less than 25 clusters. Please refer to the manuscript for further details (REF).
- It is important that the image contains well-defined clusters that can be thresholded in a meaningful way. The user should test different thresholds to see which one works best. We suggest testing the Otsu followed by the Default.

Interaction Factor Simulations Plugin

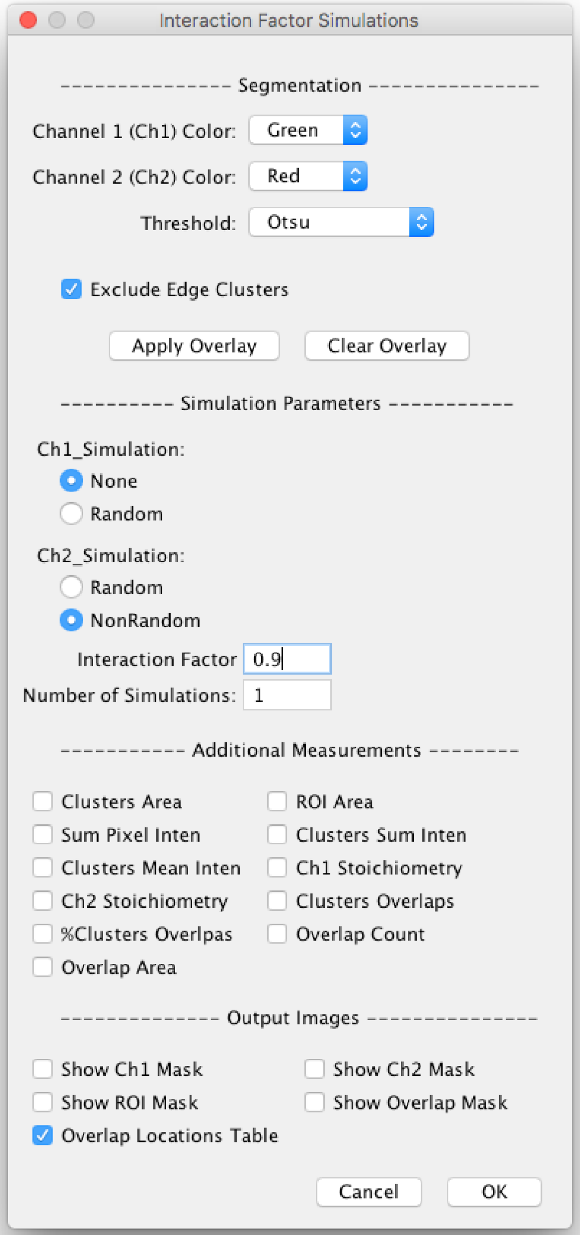

Figure 12. Interaction Factor Simulations Plugin

This plugin allows the user to create a set of simulated images at a chosen IF based on the input image. The interface for processing the input image is similar to the "**Interaction Factor**" calculation dialog box. The ***Segmentation*** and ***Additional Measurement*** sections are the same for both the IF calculation and the IF Simulation. Instead of the IF Parameter Section as in the IF Calculation, there is a ***Simulations Parameters*** section.

In this section, parameters for input into the simulation may be chosen and includes the following:

1. **Ch1 Simulation-** or Channel 1, you may chose either ***Random*** or ***None*** (default) by clicking the radio button. If ***Random*** is selected, then Ch1 clusters are placed in random locations within the ROI. If ***None*** is selected Ch1 clusters will be kept in the same position as the input image.
2. **Ch2 Simulation-** for Channel 2, you may chose either ***Random*** or ***NonRandom*** by clicking the radio button. If ***Random*** is selected, then Ch2 clusters are placed in random locations within the ROI. If ***NonRandom*** is selected, then Ch2 clusters are placed according to the IF value within the ROI.
3. **Interaction Factor-** Unlike the IF Calculation function, which gives an Interaction Factor, you must choose the Interaction Factor for this function. It is the number representing the interaction between the two color clusters. It ranges from 0 to 1 where 0 indicates no interaction and 1 indicates complete interaction.
4. **Number of Simulations-** Here you enter the number of simulations you want generated.

Running the Simulation or Simulations

Running a simulation is similar to running a calculation. As in the IF Calculation option, you must do the following to set up the simulation.

1. Open an image for analysis.
2. Using one of the selection tools, choose what ROI will be used. If the ROI covers the entire field of view there is no need for drawing an ROI.
3. In the ***Segmentation*** section, select the options you need for the input. This includes:
    a. Channel 1 Color
    b. Channel 2 Color
    c. Threshold
    d. Exclude Edge Clusters
4. Click "***Apply Overlay"***
5. Next, choose any input parameters in the ***Simulation Parameters, Additional Measurements****, and* ***Output Images*** section. Once all the options have been chosen click the "***OK"*** button and the simulation executes. The input panel will disappear and the output will be

displayed on your screen. If you decide not to execute the simulation then click the "***Cancel***" button.

Output

Each simulation will be displayed with the corresponding number at the end of the name.

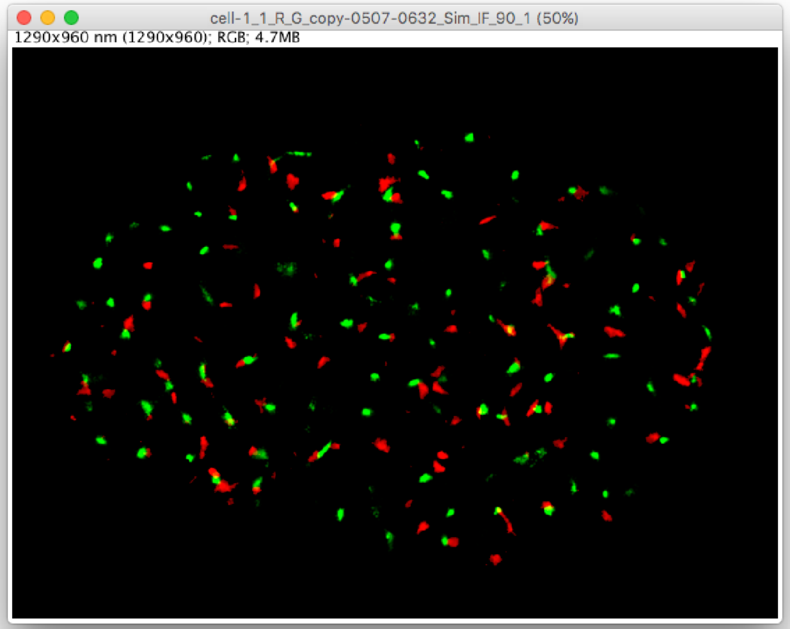

Figure 13. Example of Simulation Generated with IF = 0.90

Next, optional output masks will be displayed (refer to the previous section for details on the output masks). In addition, there will be a **Results Table** with several measurements (refer to the previous section for details on the columns). For each simulation the measurements will be added to the corresponding rows of the table.

Results

Image	Scale	G Sim	R Sim
cell-1_1_R_G_copy-0507-0632	20.0 nm	None	None
cell-1_1_R_G_copy-0507-0632_Sim_1	20.0 nm	None	NonRandom
cell-1_1_R_G_copy-0507-0632_Sim_2	20.0 nm	None	NonRandom
cell-1_1_R_G_copy-0507-0632_Sim_3	20.0 nm	None	NonRandom

Figure 14. Example of Results Table
